# Metacognitive awareness in music education: an empirical investigation of personal, educational, and practice-related correlates

**DOI:** 10.3389/fpsyg.2026.1994193

**Published:** 2026-10-28

**Authors:** Şehriban Koca, Aslıhan Bayram, Ezgi Erdoğan, Ahmet Uğur Yenigün, Alican Gülle, Melikşah Sandıkçı, Aslıhan Ergin

**Affiliations:** 1 Department of Musicology, Faculty of Music and Performing Arts, Mersin University, Mersin, Türkiye; 2 Institute of Educational Sciences, Mersin University, Mersin, Türkiye

**Keywords:** cognitive processes, correlates, instrument practice duration, knowledge of cognition, metacognition, music education, regulation of cognition

## Abstract

**Background:**

Metacognitive awareness, comprising knowledge of cognition and regulation of cognition, is theorized to be central to self-regulated learning and performance, yet evidence on how it is distributed across personal, educational, and practice-related characteristics in music education, and whether it predicts self-reported achievement, remains scarce.

**Aim:**

This descriptive-correlational study examined the metacognitive awareness profile of students receiving music education and tested whether gender, age, school type, duration of music education, instrument type, and daily practice duration were statistically associated with metacognitive awareness, and whether its two dimensions independently predicted self-reported music course achievement.

**Methods:**

Participants were 1,000 students, predominantly aged 10 to 20, receiving music education, predominantly through compulsory school curricula, in central Mersin, Türkiye, as a convenience sample. Data were collected with the Metacognitive Awareness Inventory and a demographic/practice-history form. Independent-samples *t*-tests, one- and two-way ANOVAs (including a gender-by-age-band model), the Kruskal-Wallis H test, correlations, and hierarchical regression were used, with effect sizes reported throughout.

**Results:**

Students reported moderately high metacognitive awareness overall. Female students scored significantly higher than male students on regulation of cognition and total awareness, with small, age-invariant effect sizes. Age and school type each showed a non-monotonic, U-shaped pattern, with the middle category of each (ages 15–18; Middle School) scoring lowest. Duration of music education was not significantly associated with metacognitive awareness, although students receiving private instruction scored significantly higher than those in state-curriculum classes; instrument type and daily practice duration each showed small, significant effects on overall awareness. Knowledge of cognition and regulation of cognition were moderately correlated. In hierarchical regression, only knowledge of cognition remained a significant predictor of self-reported music course achievement after controlling for age, education duration, and practice duration.

**Conclusion:**

Metacognitive awareness in music education appears shaped primarily by gender, age, and practice-related factors rather than cumulative training duration, and its knowledge dimension carries independent, if modest, predictive value for achievement, suggesting declarative/conditional self-knowledge as a pedagogical focus warranting further validation rather than a strong instructional target. Findings are correlational, based on a convenience sample and self-report measures, and should be interpreted as hypothesis-generating rather than confirmatory.

## 1 Introduction

Learning is increasingly defined not by what is acquired but by whether learners know how to regulate their own learning (Çakıroğlu, 2007). Metacognition—the awareness and control an individual exercises over their own cognitive processes (Flavell, 1979)—is central to this shift: when learners understand how they themselves learn, they analyze problems more effectively and manage the learning process more efficiently (Prescott, 2001, as cited in Koca and Çakır, 2024). It thus functions not only as an academic success factor but as a self-regulatory mechanism that structures how new knowledge is processed (Schraw and Moshman, 1995; Efklides, 2011).

Contemporary education aims to cultivate autonomous learners who plan, monitor, and evaluate their own learning rather than passively receiving it (Veenman et al., 2006). Students with high metacognitive awareness set a strategic roadmap toward their goals (Zimmerman, 2000), monitor their own performance as if from an external vantage point, and adjust strategies when discrepancies arise (Nelson and Narens, 1990)—turning learning into a disciplined, purposeful process rather than a random effort (Efklides, 2006).

Music education extends this logic into a domain where technical and psychomotor skill must be integrated with sustained attentional control (Hallam, 2001), yet how these metacognitive processes are shaped by specific personal, educational, and practice-related factors remains poorly understood. Metacognition here operates as the mechanism that turns mechanical repetition into deliberate practice, through which learners select and adapt strategies to address technical challenges (Benton, 2013; Lehmann and Ericsson, 1997), ultimately shaping both learning outcomes and performance quality (McPherson and Zimmerman, 2011; Schraw and Dennison, 1994).

### 1.1 Theoretical and conceptual framework

#### 1.1.1 Metacognition

Metacognition is defined as the ability of “thinking about thinking” that enables the individual to manage their own mental processes and forms the basis of factual knowledge, conceptual understanding, and organizational skills necessary for successful learning (Araújo et al., 2024). In the literature (Bell et al., 2023), this concept is defined as a mechanism that enables the student to become an active designer of their own learning process, rather than passively receiving information. This active design role in the learning process is based on the coordinated functioning of certain mental layers rather than a random progression. In this context, metacognition is generally examined in the literature on the axis of fundamental components that focus on awareness of cognitive processes and the control of these processes.

#### 1.1.2 Components of metacognition

In the literature, metacognition appears in two main dimensions: knowledge of cognition and regulation of cognition (Schraw et al., 2012) (see Figure 1).

**Figure 1:**
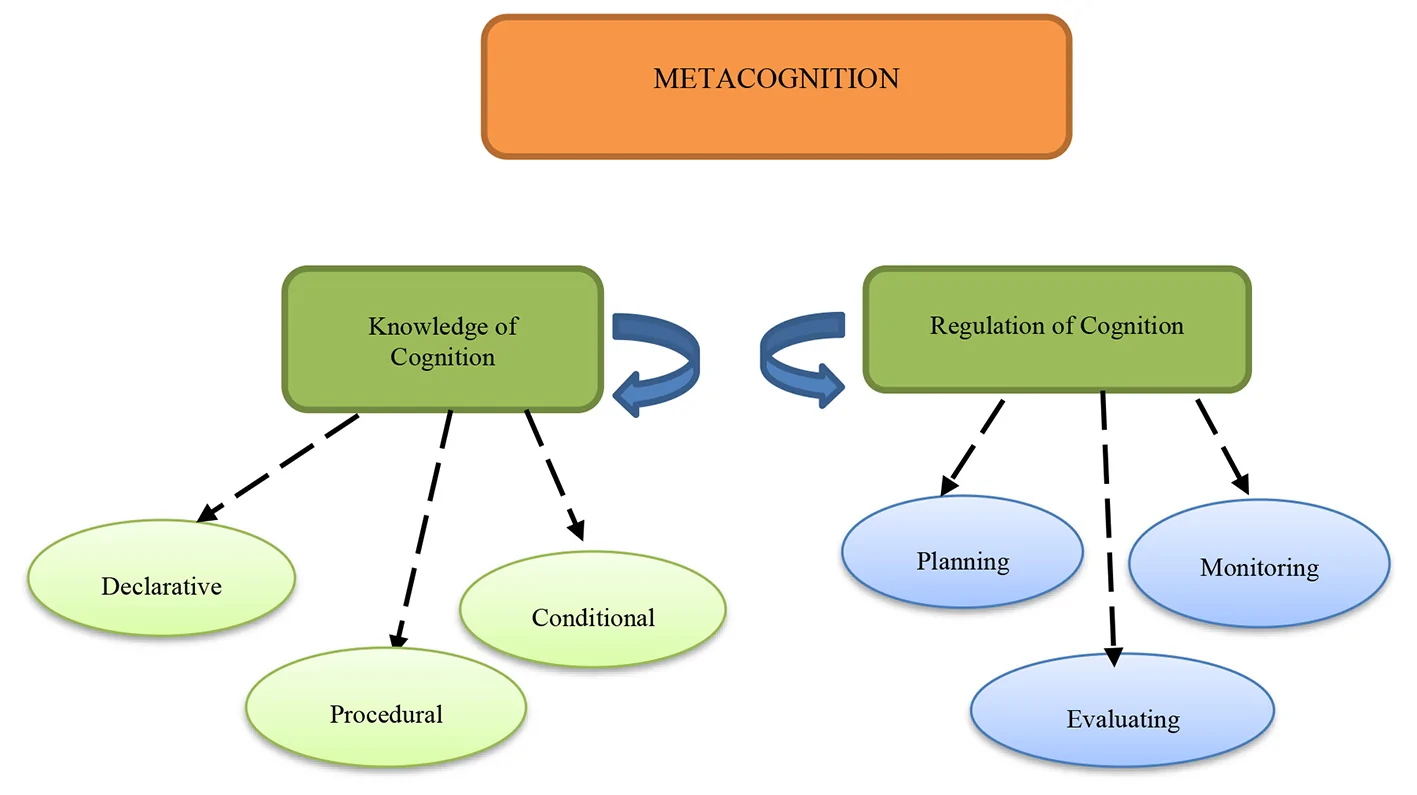
Two-dimensional structure of metacognition, adapted from Schraw and Dennison (1994), showing the knowledge of cognition (declarative, procedural, and conditional knowledge) and regulation of cognition (planning, monitoring, and evaluating) dimensions.

##### 1.1.2.1 Knowledge of cognition

It is the individual's awareness of their own cognitive capacity as a learner (Araújo et al., 2024; Li et al., 2023a). This dimension includes declarative knowledge (what one knows), procedural knowledge (how to apply strategies), and conditional knowledge (why and when to use strategies) (Mullens, 2025; Najafzade et al., 2026). For a music student, this refers to knowing which passage is technically difficult and understanding which practice method (for example, slow practice) to choose in order to overcome this difficulty (Rumiantsev et al., 2023; Veenman, 2012).

##### 1.1.2.2 Regulation of cognition

This dimension, which includes the active control of cognitive processes, consists of the cycle of planning (setting goals), monitoring (instant control of performance), debugging (changing strategies), and evaluation (analysis of results) (Vogel, 2020; Wang et al., 2025). Especially in instrument practice, the “plan–implement–reflect” cycle directly determines the student's rate of development (Zhang, 2025). Musicians' regulation of cognition encompasses not only declarative awareness but also the real-time self-monitoring of performance and the evaluative debugging of technical errors as they occur; musical feedback training has been shown experimentally to directly strengthen these monitoring and evaluative capacities (Li et al., 2023a).

In music, this awareness is considered a strong predictor of academic and performance success (Veenman et al., 2006), since musical development requires the synchronization of subcomponents such as note reading, rhythmic sense, technical execution, and expressive interpretation.

#### 1.1.3 Metacognition in musical learning

In the context of music education, metacognition is accepted as an integral dimension of musical thinking and establishes a bridge between factual knowledge and organizational skills required for successful performance or theoretical learning (Bell et al., 2023; Ferenc, 2016). These skills, referred to as “metacomponents” in Sternberg's (1985) triarchic theory of intelligence, include high-level executive processes such as planning, monitoring, and evaluation and constitute the basis of intelligent behavior (Jauk et al., 2013; Li et al., 2023a).

Successful musicians rely on strategic planning and continuous self-monitoring rather than unconscious repetition; mechanical, uncorrected repetition among beginners instead reinforces errors (Benton, 2014). Lehmann and Ericsson's (1997) concept of deliberate practice—goal-oriented, systematic, and feedback-driven—captures this distinction, and advanced musicians show a superior ability to evaluate task complexity and select optimal practice routines (Concina, 2019). Hallam (2001, p. 27) similarly defines effective instrumental practice as practice that reaches the desired outcome in the shortest time, requiring efficient time use, sustained concentration, and motivation. Learning an instrument is thus governed as much by how a student practices as by what is played: questions such as “why can I not play this passage?” or “which practice method solves this technical problem?” are themselves expressions of metacognitive awareness, the mechanism through which technical skill is converted into performance. Such self-monitoring does not develop in isolation: qualitative evidence from Chinese university music classrooms indicates that teachers' scaffolding—including modeling, guided listening, and timely feedback—is perceived by both teachers and students as fostering learners' autonomy and self-regulated engagement with practice (Li and Wang, 2026), suggesting that metacognitive awareness in music learning is shaped jointly by learner characteristics and instructional context.

### 1.2 Research gap

These practice-level observations point to a broader, still unresolved question: research on metacognition in music has intensified over the last two decades, establishing that musical success depends not only on technical skill but on how students monitor and regulate their own learning (McPherson and McCormick, 2006), yet this literature has rarely examined how metacognitive awareness itself is shaped by the personal, educational, and practice-related characteristics of the learners who report it, or whether it carries any demonstrable link to achievement rather than only to other self-report measures.

It is also worth noting that findings across this literature are not always consistent in magnitude or interpretation: deliberate-practice theory (Ericsson et al., 1993) predicts that mere accumulated duration should not, by itself, build metacognitive skill without structured, effortful, feedback-driven engagement, whereas simpler comparative designs in the literature sometimes report very large differences between trained and untrained groups without controlling for this distinction, which can give a misleading impression of a straightforward, linear dose–response relationship. Domain-specific instruments have also begun to emerge in response to the limitations of general academic metacognition scales in this context; Li et al. (2023b), for instance, developed an eight-factor Metacognitive Questionnaire for Music Learning (MQML) that more directly captures the dynamics of instrumental practice. The present study nonetheless retains the more established, general-education MAI/Jr. MAI framework (Aydın and Ubuz, 2010) rather than a music-specific instrument such as the MQML, in order to permit direct comparison with the broader metacognition literature; this trade-off is revisited in the Limitations. Two specific limitations follow from this broader gap. First, existing quantitative work leaves the differentiating role of variables such as gender, age, school type, instrument played, and daily practice duration insufficiently modeled jointly, particularly in the Turkish context, and is often limited by small, low-power samples. Second, and more fundamentally, this literature has relied almost exclusively on self-report measures correlated with other self-report or demographic variables, without testing whether metacognitive awareness relates to any self-reported achievement outcome; it remains unknown whether metacognitive awareness, and if so, which of its two dimensions, predicts self-reported music-course achievement once age and experience-related variables (duration of education, daily practice) are statistically controlled. This second question carries direct pedagogical weight: if neither dimension predicts achievement beyond experience, interventions targeting metacognitive awareness would have limited justification; if one does, this identifies a specific, trainable instructional target. The present study addresses this gap directly (RQ5) via hierarchical regression testing whether knowledge of cognition and regulation of cognition each contribute unique predictive variance beyond age, duration of music education, and daily practice duration.

Building on a large sample of 1,000 participants, this study's distinct contribution is threefold: (a) to our knowledge, it is the first to jointly model demographic (gender, age), educational (school type, grade level, duration), and practice-related (instrument type, daily practice) correlates of metacognitive awareness within a single large-scale sample of students receiving music education in the central districts of Mersin, Türkiye, spanning primary through secondary educational levels, rather than examining these variable classes in isolation; (b) it combines parametric and non-parametric approaches to accommodate the unbalanced subgroup sizes typical of convenience samples; and (c) it reports quantified effect sizes throughout, allowing readers to judge practical as well as statistical significance. The guiding research question is: what is the level of metacognitive awareness among students receiving music education, and how does it vary according to personal, educational, and practice-related characteristics? Findings are expected to contribute an evidence-based account of how metacognitive awareness is distributed across music education contexts in the central districts of Mersin, Türkiye, and to inform pedagogical practice.

### 1.3 Objectives and research questions

The present study aims to systematically investigate the baseline profile and internal structure of metacognitive awareness—specifically knowledge of cognition and regulation of cognition—among students receiving music education. Grounded in a domain-specific cognitive individual differences perspective, metacognitive awareness is positioned as a key self-regulatory construct in learning, while students' demographic backgrounds, educational settings, and active musical practice habits are analyzed as interacting factors that shape this cognitive development.

Drawing on a large sample of music education students in the central districts of Mersin, Türkiye (*N* = 1,000), the study seeks to clarify how these diverse personal and contextual variables interact with metacognitive capabilities. Rather than proposing an overly complex, non-linear structural causal design, the investigation deliberately adopts a theoretically driven and parsimonious analytical approach. This ensures that the evaluation of group differences and correlations remains statistically transparent and strictly proportionate to the cross-sectional evidence, aligning with rigorous standards for survey-based cognitive research.

Within this framework, the primary objective is to map the overarching metacognitive competencies of music students, while explicitly examining the variance explained by specific educational and practical background metrics. To achieve this purpose, the study addresses the following clear research questions:

RQ1. What is the general descriptive profile of metacognitive awareness (knowledge of cognition and regulation of cognition) among students receiving music education, and do these two dimensions correlate moderately to strongly with one another while remaining empirically distinguishable, consistent with Schraw and Dennison's (1994) two-dimensional structure?
RQ2. Do students' levels of metacognitive awareness differ significantly according to (a) gender, with female students expected to show higher awareness, particularly in regulation of cognition, consistent with documented gender differences in self-regulatory behavior (Pintrich and De Groot, 1990; Pajares and Valiante, 1997), albeit with a modest effect size, and (b) age, explored here as an open question given the absence of prior population-specific evidence: does awareness vary non-monotonically across age, with the possibility of comparatively lower scores among students aged 15–18 relative to both younger and older groups, reflecting the cognitive and motivational turbulence of this developmental transition (Veenman et al., 2006; Kuhn, 2000)?
RQ3. Do students' levels of metacognitive awareness differ significantly based on educational context variables, namely (a) school type and grade level, reflecting differences in curricular intensity and cognitive demands across educational stages, and (b) total duration of music education, explored as an open question: is exposure duration alone related to awareness, or does it fail to predict awareness once the quality and intentionality of practice are considered (Ericsson et al., 1993)?
RQ4. Do students' levels of metacognitive awareness differ significantly according to music-related practice variables, namely (a) instrument type, consistent with prior evidence of domain-specific practice demands, and (b) daily practice duration, explored as an open question given the absence of established dose–response findings for this population: is this relationship linear or non-linear (e.g., an inverted-U pattern), and does it hold once the small, unbalanced subgroup sizes at higher durations are taken into account?
RQ5. Do the two dimensions of metacognitive awareness independently predict students' self-reported music course achievement when age, duration of music education, and daily practice duration are statistically controlled, with knowledge of cognition expected to show the more robust independent contribution given that declarative and conditional self-knowledge is theorized to precede behavioral regulation of practice?

## 2 Methods

### 2.1 Research model/design

This study adopts a quantitative research design to examine the metacognitive awareness of music education students in relation to both individual and contextual variables. A descriptive survey design was employed, which enables the systematic depiction of an existing situation and is particularly effective for identifying the opinions, attitudes, and abilities of large populations (Fraenkel and Wallen, 2009, p. 390).

The selection of the survey design in the present study is grounded in several methodological considerations. First, the large sample size (*N* = 1,000) enhances statistical precision and supports the examination of group differences with adequate power. It is important to note, however, that the use of convenience sampling means that findings cannot be straightforwardly generalized beyond the specific population from which the sample was drawn—namely, music education students enrolled in schools within the central districts of Mersin, Türkiye. Second, it supports both descriptive and relational analyses by enabling the examination of participants' levels of knowledge of cognition and regulation of cognition in their current form, as well as their associations with variables such as gender, school type, and instrument discipline (Karasar, 2009). Third, the use of standardized data collection procedures ensured that the research data were systematically obtained through structured self-report instruments, supporting replication and comparison with future studies. This methodological framework facilitated a coherent integration between the theoretical foundations of the study and its empirical findings, ensuring consistency between conceptual assumptions and practical outcomes.

### 2.2 Population and sample of the study

The research population encompasses students enrolled in various educational tracks—specifically Fine Arts High Schools, Science and Art Centers, and general secondary institutions—under the Ministry of National Education in Mersin, Türkiye, during the 2022–2023 academic cycle. From this population, a sample of 1,000 participants was recruited from Mersin's central districts (Toroslar, Yenişehir, Akdeniz and Mezitli). The selection process utilized a convenience sampling approach to ensure practical accessibility and diverse student representation. The reasons for preferring the convenience sampling design in this study are presented below with their academic justifications:

Accessibility and practical applicability: music education shows a heterogeneous distribution in terms of institutional structure and diversity of practices. The time, cost, and transportation constraints encountered in accessing this target group spread over a wide geographical area necessitated the researcher to select these units through which data could be obtained in the fastest and most reliable way (Fraenkel et al., 2012, p. 99).

Sample size and statistical power: although the convenience sampling method is generally associated with the risk of limited generalizability, the sample size reached in this study (*N* = 1,000) provides sufficient statistical power to detect meaningful group differences within the specific institutional and geographic scope of the study. Therefore, these results primarily characterize the metacognitive profiles of music students in the central districts of Mersin and should not be directly generalized to the entire national population of music students in Türkiye (Creswell and Guetterman, 2019). Findings should be interpreted within this geographic and institutional scope to maintain statistical precision while acknowledging the constraints of the sampling design.

Voluntariness and data quality: due to the sensitivity of education processes based on music performance, the voluntariness of participants and the openness of institutions to cooperation were prioritized during the data collection phase. In this direction, including educational institutions that provided ease of access to the researcher and offered implementation support in the sample maximized the response rate by preventing data loss (Patton, 2015, p. 264).

### 2.3 Data collection tools

#### 2.3.1 Personal information form

The personal information form used in the study includes questions created to obtain information such as age, gender, educational status, the school attended, the type of school attended, the type of institution where music education is received, grade level, duration of music education, how many hours per week music education is received, which instrument they play, how many hours on average they practice per day, the music course achievement score range, and the overall academic achievement score range at school. Among these, the music course achievement score range (self-reported on a five-band ordinal scale: 0–44, 45–54, 55–69, 70–84, and 85 and above, corresponding to standard Turkish school grading bands) is used in this study as an outcome measure of musical competence, allowing the predictive, rather than purely descriptive, relevance of the two metacognitive awareness dimensions to be tested directly (RQ5). While this is a coarse, self-reported proxy rather than a standardized performance assessment, it is, to our knowledge, the only available achievement-related criterion in this dataset, and its inclusion allows the study to move beyond demographic correlates toward criterion-related predictive evidence. Age was recorded through three pre-defined age-range categories (10–14, 15–18, and 19+ years) rather than as a continuous variable; consequently, the descriptive and inferential statistics reported for age reflect these categorical bands rather than exact chronological age. Regarding the status of music education, the majority of participants (*n* = 874) were enrolled in state schools, where music education is delivered as part of the mandatory national curriculum, while a smaller subgroup (*n* = 124) received music instruction through private course centers on a voluntary, fee-based basis; this distinction is examined directly as a supplementary analytic variable in Section 3.5.1, given its direct relevance to interpreting the duration-of-education variable below. For consistency, this article uses “general school music education” for compulsory, state-curriculum classroom instruction and “private or specialized instruction” for fee-based course-center training. Age and grade level are treated as conceptually and empirically distinct variables in this study: age reflects a student's chronological/developmental stage, whereas grade level reflects institutional placement within the formal curriculum. Although the two are correlated in most educational systems, they are not interchangeable, since students of the same age may be enrolled in different grades (e.g., due to grade retention/acceleration, differing school-entry ages, or enrollment in mixed-age tracks such as some vocational or private course-based music programs), and students in the same grade may span more than one age band; modeling them separately allows the study to disentangle developmental (age-related) effects from institutional/curricular (grade-related) effects on metacognitive awareness, rather than assuming the two are redundant.

#### 2.3.2 Metacognitive awareness inventory (MAI)

Data regarding students' metacognitive levels were gathered using the MAI originally developed by Schraw and Dennison (1994) and later adapted for younger populations by Sperling et al. (2002), with the present 17-item form adapted into Turkish by Aydın and Ubuz (2010). The Turkish version's psychometric properties were previously validated for the target cultural context. This 17-item instrument employs a five-point Likert scale, ranging from ‘never' (1) to ‘always' (5), yielding a total possible score range of 17–85, with higher scores reflecting greater metacognitive awareness. The knowledge of cognition sub-dimension comprises eight items (items 1–5 and 11–13; possible range: 8–40) and the regulation of cognition sub-dimension comprises nine items (items 6–10 and 14–17; possible range: 9–45). According to the adaptation study by Aydın and Ubuz (2010), the internal consistency coefficients were reported as α = 0.75 for the knowledge sub-dimension and α = 0.79 for the regulation sub-dimension. In the present sample (*N* = 1,000), Cronbach's alpha coefficients were calculated as α = 0.76 for knowledge of cognition, α = 0.81 for regulation of cognition, and α = 0.84 for the total scale, indicating adequate to good internal consistency and confirming the reliability of the instrument in this specific sample. These values are consistent with those reported in the original adaptation study and support the appropriateness of the two-dimensional structure of the MAI for the present context. It should be noted that the present study rechecked only the internal consistency of the previously validated Turkish MAI structure and did not re-conduct a confirmatory factor analysis (CFA) on the current sample; reliability evidence alone should not be interpreted as structural validity evidence. The two-dimensional structure is instead adopted on the basis of the original validation by Aydın and Ubuz (2010), who conducted both EFA (*N* = 314) and CFA (*N* = 589) on independent Turkish-school samples and reported a good-fitting two-factor model (χ^2^(99) = 285.71, χ^2^/df = 2.88, RMR = 0.05, GFI = 0.94, AGFI = 0.92, RMSEA = 0.05, CFI = 0.91) that fit significantly better than both a one-factor and a null model (Δχ^2^ = 438.35 and 150.73, respectively, both *p* < 0.001), with subscale reliabilities of α = 0.75 (knowledge) and α = 0.79 (regulation) and an inter-subscale correlation of *r* = 0.72. This prior, independently CFA-validated structure provides the structural-validity basis for the two-dimensional scoring adopted here.

### 2.4 Data collection process

In the data collection process of the study, firstly, the required ethical approval was obtained by the decision of the Mersin University Social and Humanities Ethics Committee (dated 12.07.2021 and numbered 54). Permissions were obtained from the Mersin Provincial Directorate of National Education, and the data collection process was initiated by obtaining consent from the parents of the students who constituted the sample of the study through a parental consent form.

### 2.5 Data analysis

All statistical analyses were conducted using IBM SPSS Statistics. Before the analysis process, checks for erroneous data entry and outliers were conducted on the data. In the selection of hypothesis tests, the distribution characteristics of the data, the homogeneity of variances, and group sizes were taken as the basis. The procedures followed and the justifications for test selection in the analysis phase are presented below.

Normality and parametric tests: considering the large sample size (*N* = 1,000) and the central limit theorem, skewness and kurtosis coefficients were confirmed to fall within the range of ± 1.5 to ± 2 (Tabachnick and Fidell, 2013, p. 79). In light of these findings, the Independent Samples *t*-test was applied for the analysis of differences according to the gender variable, and one-way analysis of variance (ANOVA) was applied for variables with three or more categories, such as age and school level (Field, 2013, p. 171). For inferential analyses, school type was collapsed into three levels (Primary/Middle/High School), with the five high-school subtypes combined into a single “High School” category, and duration of music education was collapsed into three levels, with the 5–6 and 7+ year groups combined into a single “5+ years” category (Table 1), to ensure adequate subgroup sizes for ANOVA.

**Table 1:** ANOVA results of metacognitive dimensions and global awareness scores according to the duration of music education.

Sub-dimensions	Duration of music education (year)	*N*	M	SD	95% CI	*F*	*p*	$\eta_{p}^{2}$
Knowledge of cognition	1	346	32.55	4.63	[32.06–33.04]	2.23	0.11	0.004
	2–4	363	32.72	4.57	[32.25–33.19]			
	5+	291	33.30	4.74	[32.76–33.84]			
Regulation of cognition	1	346	32.08	6.32	[31.41–32.75]	1.69	0.18	0.003
	2–4	363	32.39	6.34	[31.74–33.04]			
	5+	291	33.00	6.39	[32.27–33.73]			
Metacognitive awareness	1	346	64.64	9.73	[63.61–65.67]	2.37	0.09	0.005
	2–4	363	65.12	9.73	[64.12–66.12]			
	5+	291	66.31	10.07	[65.15–67.47]			

All models are evaluated with an analytical degree of freedom configuration of df (2, 997). Significance threshold is set at *p* < 0.05.

Non-parametric tests: for the variable of daily instrument practice duration, the assumption of homogeneity of variances was violated (Levene *p* < 0.05) and group sizes were extremely unbalanced (e.g., those practicing 5 h or more, *n* = 6). Given the risk of parametric tests producing erroneous results under these conditions, the Kruskal–Wallis H test, which provides a more flexible approach, was preferred instead (Pallant, 2020, p. 224).

Multiple comparisons (*post-hoc*): to mitigate the risk of Type I errors associated with multiple testing, *post-hoc* comparisons were conducted using two decision-based procedures selected according to Levene's test of homogeneity of variance: Tukey's HSD was applied when Levene's test was non-significant (*p* > 0.05, indicating equal variances across groups), and Tamhane's T2 was applied when Levene's test was significant (*p* ≤ 0.05, indicating unequal variances), following Field (2013, p. 460). This variance-dependent selection was adopted to maintain statistical power and control the family-wise error rate across all sub-dimensions, regardless of their homogeneity status (Creswell and Guetterman, 2019).

As a robustness check, the six pairwise comparisons for daily practice duration (Table 2) were additionally evaluated using the Holm-Bonferroni step-down procedure. This yielded identical conclusions to the standard Bonferroni correction reported above, with only the “not practicing vs. 1 h” contrast remaining significant; the findings are therefore robust to the choice of multiple-comparison correction.

**Table 2:** Kruskal–Wallis *H*-test results of metacognitive dimensions and global awareness scores according to daily practice duration.

Sub-dimensions	Daily instrument practice duration	Mean ranks	95% CI^a^	SD	χ^2^	*p*
Knowledge of cognition	Not working	475.95	—	4.65	21.757	0.000^*^
	1 h	567.83	—			
	2 to 4 h	655.55	—			
	5+	485.75	—			
Regulation of cognition	Not working	479.49	—	6.35	15.843	0.01^**^
	1 h	556.36	—			
	2 to 4 h	646.55	—			
	5+	538.67	—			
Metacognitive awareness	Not working	475.35	—	9.84	22.660	0.000^*^
	1 h	568.39	—			
	2 to 4 h	668.60	—			
	5+	514.67	—			

^*^p = 0.001, ^**^p = 0.01. ^a^95% confidence intervals for mean ranks are not reported, as standard CI formulas developed for arithmetic means are not directly applicable to rank-based statistics and produced implausibly narrow intervals; see Bonferroni-adjusted pairwise comparisons reported in the text below.

Relational analyses: Pearson product-moment correlation analysis was conducted to determine the structural relationships between the scale sub-dimensions and total score, with the interpretation of coefficients based on the criteria suggested by (Schraw and Dennison 1994, p. 468) and Miles and Shevlin (2001, p. 115).

Effect size: beyond statistical significance (*p*) alone, Cohen's d for the *t*-test, eta-squared (η^2^) for ANOVA, and epsilon-squared values for the Kruskal–Wallis test were calculated to determine the practical significance of the findings (Ellis, 2010, p. 41). In order to ensure statistical transparency and provide a more precise estimation of the observed differences, 95% confidence intervals (CIs) were also reported consistently across all inferential analyses (Tables 1–6). The inclusion of CIs alongside exact *p*-values and effect sizes (Cohen's d, η^2^, and ε^2^) allows for a rigorous assessment of the precision of group means and the stability of the findings, particularly in instances involving unbalanced subgroups; this multi-metric approach ensures that the interpretation of results is driven not only by statistical significance but also by the practical magnitude and precision of the evidence.

**Table 3:** *T*-test results of metacognitive dimensions and global awareness scores according to gender.

Sub-dimensions	Gender	*N*	M	SD	95% CI	*t*	*p*	Cohen's *d*
Knowledge of cognition	Female	578	33.03	4.44	[32.67–33.39]	1.48	0.14	0.09
	Male	420	32.59	4.89	[32.12–33.06]			
Regulation of cognition	Female	578	32.86	6.05	[32.37–33.35]	2.21	0.03^*^	0.14
	Male	420	31.96	6.71	[31.32–32.60]			
Metacognitive awareness	Female	578	65.89	9.45	[65.12–66.66]	2.13	0.03^*^	0.14
	Male	420	64.55	10.29	[63.57–65.53]			

^*^p < 0.05.

**Table 4:** ANOVA results of metacognitive dimensions and global awareness scores according to age.

Sub-dimensions	Age	*N*	M	SD	95% CI	*F*	*p*	* $\eta_{p}^{2}$ *
Knowledge of cognition	10–14	119	33.64	4.30	[32.87–34.41]	9.37	0.000^*^	0.018
	15–18	586	32.30	4.65	[31.92–32.68]			
	19+	295	33.56	4.64	[33.03–34.09]			
Regulation of cognition	10–14	119	33.68	5.71	[32.65–34.71]	12.17	0.000^*^	0.024
	15–18	586	31.64	6.55	[31.11–32.17]			
	19+	295	33.61	5.96	[32.93–34.29]			
Metacognitive awareness	10–14	119	67.32	8.85	[65.73–68.91]	13.75	0.000^*^	0.027
	15–18	586	63.94	9.98	[63.13–64.75]			
	19+	295	67.17	9.53	[66.08–68.26]			

df = (2, 997); ^*^Indicates significant *p*-value. ^***^p < 0.001.

**Table 5:** ANOVA results of metacognitive dimensions and global awareness scores according to the type of school attended.

Sub-dimensions	Type of school	*N*	M	SD	95% CI	*F*	*p*	$\eta_{p}^{2}$
Knowledge of cognition	Primary	58	34.82	3.31	[33.97–35.67]	10.32	0.000^*^	0.020
	Middle	535	32.31	4.89	[31.90–32.72]			
	High	407	33.23	4.37	[32.80–33.66]			
Regulation of cognition	Primary	58	34.17	6.10	[32.60–35.74]	4.41	0.01^**^	0.009
	Middle	535	31.98	6.55	[31.42–32.54]			
	High	407	32.85	6.08	[32.26–33.44]			
Metacognitive awareness	Primary	58	69.00	8.46	[66.82–71.18]	8.26	0.000^*^	0.016
	Middle	535	64.30	10.22	[63.43–65.17]			
	High	407	66.08	9.33	[65.17–66.99]			

df = (2, 997); ^*^p < 0.05, ^**^p < 0.01, ^***^p < 0.001.

**Table 6:** ANOVA results of metacognitive dimensions and global awareness scores according to the type of instrument played.

Sub-dimensions	Instrument	*N*	M	SD 95% CI	*F*	*p*	η^2^
Knowledge of cognition	Keyboard	205	33.21	4.04 [32.66–33.76]	8.56	0.000^*^	0.046
	String	56	34.67	4.92 [33.38–35.96]			
	Wind	281	33.62	4.05 [33.15–34.09]			
	Bowed string	52	33.25	4.02 [32.16–34.34]			
	Percussion	123	32.21	4.60 [31.45–33.61]			
Regulation of cognition	Keyboard	205	33.54	5.75 [32.75–34.33]	1.51	0.20	0.008
	String	56	33.23	6.08 [31.64–34.82]			
	Wind	281	33.19	5.78 [32.51–33.87]			
	Bowed string	52	33.51	4.96 [32.16–34.86]			
	Percussion	123	32.53	6.13 [31.45–33.61]			
Metacognitive awareness	Keyboard	205	66.76	8.67 [65.57–67.95]	3.39	0.01	0.019
	String	56	67.91	8.91 [65.58–70.24]			
	Wind	281	66.82	8.85 [65.79–67.85]			
	Bowed string	52	66.76	8.01 [64.58–68.94]			
	Percussion	123	62.18	9.62 [60.48–63.88			

^*^Indicates significant *p*-value. ^***^p < 0.001. A total of 283 cases who did not specify their instrument discipline were excluded from this specific sub-analysis, resulting in an active sample size of *N* = 717 with a model degree of freedom of df (4, 712).

Predictive analysis: to test RQ5, a hierarchical multiple linear regression was conducted with music course achievement (five-band ordinal outcome, treated as an approximately interval-scaled criterion given the large sample size, following common practice for coarse ordinal outcomes with *N* > 30 per category) (Norman, 2010) as the dependent variable. Age, duration of music education, and daily practice duration were entered as covariates, followed by the two standardized metacognitive awareness sub-dimension scores (knowledge of cognition and regulation of cognition) as simultaneous predictors, allowing their independent, incremental contribution to be estimated net of experience-related variables.

Sensitivity and robustness analyses: several additional analyses were conducted to evaluate the robustness of the main findings. First, the three-band age grouping used in the primary analyses was cross-checked against a finer five-band grouping (10–13, 14–15, 16–18, 19–20, 21+) derived directly from the raw age responses. Second, following a request to examine whether gender effects vary developmentally rather than treating age only as a linear covariate, a 2 (gender) × 3 (age band: 10–14, 15–18, 19+) factorial ANOVA was conducted for each metacognitive awareness dimension, testing the main effects of gender and age band together with their interaction (results reported in Table 7). Third, a joint regression model entering age band, school type, and weekly hours of music instruction simultaneously was used to evaluate whether apparent age- and school-type-related differences might instead be attributable to curricular intensity (results reported in Table 8); grade level was excluded from this joint model due to its high collinearity with age (*r* = 0.95). Fourth, given that the achievement outcome used in the hierarchical regression (Section 2.3.1) is a five-band ordinal self-report, an ordinal logistic regression was estimated as a sensitivity analysis for the hierarchical regression reported under RQ5. All sensitivity analyses are reported alongside their corresponding primary results in the Discussion. Fifth, because institution type (state/mandatory vs. private/fee-based music instruction; Section 2.2) was initially treated only as sample background, an independent-samples Welch *t*-test was conducted *post hoc* to test directly whether it is associated with metacognitive awareness, together with chi-square tests of association between institution type and both duration of music education and daily practice duration, to clarify what the duration variable does and does not capture (reported in Section 3.5.1). Sixth, to test whether the practice-duration finding (RQ4b) depends on the fine-grained four-category coding, a simplified binary contrast between students reporting any regular daily practice and those reporting none was evaluated with a Welch *t*-test (reported in Section 3.7). Seventh, because two of the four daily-practice subgroups are very small (2–4 h: *n* = 10; 5+ h: *n* = 6), three assumption-light procedures were added to evaluate whether the practice-duration findings depend on asymptotic approximations: bias-corrected and accelerated (BCa) bootstrap confidence intervals (10,000 resamples) for each subgroup mean; two-sided permutation tests (100,000 resamples) for all pairwise mean differences among the four subgroups, as a small-sample alternative to the asymptotic Mann–Whitney/Bonferroni procedure reported above; and a Jonckheere–Terpstra test for an overall ordered trend across the four naturally ordered practice categories (none < 1 h < 2–4 h < 5+ h), which pools information across all subgroups rather than relying on any single pairwise comparison and is therefore better powered when some categories are small. Results are reported alongside the primary findings in Section 3.7.

**Table 7:** Two-way ANOVA (Gender × age band) results for metacognitive awareness dimensions (*N* = 995).

Dimension	Effect	*F*	df	*p*	partial η^2^
Knowledge of cognition	Gender	2.60	1, 989	0.107	0.003
Knowledge of cognition	Age band	8.86	2, 989	< 0.001	0.018
Knowledge of cognition	Gender × age band	0.89	2, 989	0.409	0.002
Regulation of cognition	Gender	5.11	1, 989	0.024	0.005
Regulation of cognition	Age band	12.97	2, 989	< 0.001	0.026
Regulation of cognition	Gender × age band	0.59	2, 989	0.553	0.001
Total metacognitive awareness	Gender	4.91	1, 989	0.027	0.005
Total metacognitive awareness	Age band	13.85	2, 989	< 0.001	0.027
Total metacognitive awareness	Gender × age band	0.49	2, 989	0.611	0.001

Age band: 10–14, 15–18, 19+ (as in Table 4). partial η^2^ = partial eta-squared. The Gender × Age Band interaction was not significant for any dimension, indicating that the gender difference in regulation of cognition and overall awareness is of similar magnitude across all three age bands.

**Table 8:** Joint regression model predicting overall metacognitive awareness from age band, school type, and weekly hours of music instruction (*N* = 1,000).

Predictor	B	SE	*t*	*p*	95% CI
Intercept	63.886	1.585	40.32	< 0.001	[60.78, 67.00]
Age band: 15–18 (vs. 10–14)	−1.821	1.245	−1.46	0.144	[−4.27, 0.62]
Age band: 19+ (vs. 10–14)	1.673	1.637	1.02	0.307	[−1.54, 4.89]
School type: middle (vs. high)	1.093	1.011	1.08	0.280	[−0.89, 3.08]
School type: primary (vs. high)	4.168	1.944	2.14	0.032	[0.35, 7.98]
Weekly hours of music instruction	0.656	0.241	2.72	0.007	[0.18, 1.13]

*R*^2^ = 0.038, *F*_(5, 994)_ = 7.95, p < 0.001. Reference categories: age band 10–14; school type High School. Grade level was not entered in this joint model because of high collinearity with age (r = 0.95, VIF >10). Dependent variable: overall metacognitive awareness (sum score, range 17–85).

## 3 Results

This section details the descriptive statistics for the primary variables and explores the inter-relationships among the scale's sub-dimensions. Table 9 summarizes the descriptive metrics for both the sub-scales and the overall ‘metacognitive awareness' scores, including skewness and kurtosis coefficients utilized to assess distributional normality. Furthermore, the associations between the scale's total and sub-dimension scores, determined via Pearson product-moment correlation analysis, are reported below.

**Table 9:** Descriptive statistics and normality values related to metacognitive awareness scores.

Sub-dimensions	*N*	Min.	Max.	M	SD	Skewness	Kurtosis
“Knowledge of cognition”	1,000	8.0	40.0	32.8	4.65	−0.52	1.72
“Regulation of cognition”	1,000	9.0	45.0	32.4	6.36	−0.30	0.37
“Metacognitive awareness”	1,000	17.0	85.0	65.3	9.84	−0.44	0.27

### 3.1 Correlational structure of metacognitive awareness (RQ1)

As shown in Table 9, skewness and kurtosis coefficients for all sub-dimensions fell within the acceptable ± 2.0 interval, which is indicative of an approximately normal distribution according to commonly adopted criteria in the social sciences and psychometric literature (George and Mallery, 2010; Tabachnick and Fidell, 2013). Given the relatively large sample size (*N* = 1,000), this normality supports the suitability of applying parametric statistical methods, including *t*-tests and ANOVA, for the subsequent comparative analyses aligned with the study's sub-objectives.

Pearson correlation analysis reveals a positive, moderate, and statistically significant association between “knowledge of cognition” and “regulation of cognition”, the two core components of metacognition (*r* = 0.59, *p* < 0.001). The overall “metacognitive awareness” score demonstrated strong positive correlations with both sub-dimensions, showing a high correlation with “knowledge of cognition” (*r* = 0.85, *p* < 0.001) and an even stronger relationship with “regulation of cognition” (*r* = 0.92, *p* < 0.001). These correlational patterns are interpreted in Section 4.1.

### 3.2 Metacognitive awareness and gender (RQ2a)

Table 3 reports the independent samples *t*-test results utilized to evaluate whether students' metacognitive awareness and underlying dimensions exhibit significant variations based on the gender variable, addressing the second research question (RQ2).

As shown in Table 3, no statistically significant gender difference emerged for the “knowledge of cognition” sub-dimension [*t*_(996)_ = 1.48, *p* = 0.14]. In contrast, female students scored significantly higher than male students on both the “regulation of cognition” sub-dimension [*t*_(996)_ = 2.21, *p* = 0.03] and overall “metacognitive awareness” [*t*_(996)_ = 2.13, *p* = 0.03]; in both cases, however, the effect sizes were small (Cohen's *d* = 0.14), indicating limited practical significance despite statistical significance.

To evaluate whether this baseline gender difference varies developmentally or depends on age group, a 2 (gender) × 3 (age band: 10–14, 15–18, 19+) factorial ANOVA was conducted as a formal robustness check, with full results reported in Table 7. The main effect of gender remained significant for regulation of cognition, *F*_(1, 989)_ = 5.11, *p* = 0.024, partial η^2^ = 0.005, and overall metacognitive awareness, *F*_(1, 989)_ = 4.91, *p* = 0.027, partial η^2^ = 0.005, with female students continuing to score somewhat higher; the gender effect for knowledge of cognition remained non-significant, *F*_(1, 989)_ = 2.60, *p* = 0.107. The main effect of age band was significant for all three dimensions (all *p* < 0.001), mirroring the developmental pattern reported in Section 3.3 (Table 4). Critically, the Gender × Age Band interaction was not significant for knowledge of cognition, *F*_(2, 989)_ = 0.89, *p* = 0.409, partial η^2^ = 0.002, regulation of cognition, *F*_(2, 989)_ = 0.59, *p* = 0.553, partial η^2^ = 0.001, or overall awareness, *F*_(2, 989)_ = 0.49, *p* = 0.611, partial η^2^ = 0.001, indicating that the magnitude of the gender difference does not vary meaningfully across these three developmental stages.

### 3.3 Metacognitive awareness and age (RQ2b)

Table 4 presents the One-Way Analysis of Variance (ANOVA) outputs executed to determine whether students' metacognitive awareness profiles and sub-components significantly differ across distinct developmental age boundaries, addressing the requirements of the second research question (RQ2).

As shown in Table 4, the age variable was associated with statistically significant differences in the “knowledge of cognition” [*F*_(2, 997)_ = 9.37, *p* < 0.001], “regulation of cognition” [*F*_(2, 997)_ = 12.17, *p* < 0.001], and overall “metacognitive awareness” [*F*_(2, 997)_ = 13.75, *p* < 0.001] scores. *Post-hoc* Tukey HSD comparisons indicated that the 10–14 and 19+ age groups scored significantly higher than the 15–18 age group across all dimensions, producing a non-monotonic (U-shaped) pattern across the three age bands. The partial eta-squared values (η^2^*p*= 0.02–0.03) indicate that age accounts for a small proportion of the total variance in metacognitive awareness (Ellis, 2010; Fritz et al., 2012).

### 3.4 Metacognitive awareness and type of school attended (RQ3a)

Table 5 presents the One-Way Analysis of Variance (ANOVA) results conducted to explore whether students' metacognitive profiles differ significantly according to the type of school attended, addressing the institutional dimension of the third research question (RQ3).

As shown in Table 5, the type of school attended was associated with a statistically significant difference in metacognitive awareness across all three dimensions (knowledge of cognition, regulation of cognition, and overall awareness). As a result of the multiple comparison *post-hoc* Tukey HSD analyses conducted to determine the source of these differences, it is seen that in the “knowledge of cognition” dimension, the mean scores of primary school students (*M* = 34.82, *SD* = 3.31, 95% CI [33.97–35.67]) are significantly higher than those of both middle school students (*M* = 32.31, *SD* = 4.89, 95% CI [31.90–32.72]) and high school students (*M* = 33.23, *SD* = 4.37, 95% CI [32.80–33.66]). In the “regulation of cognition” sub-dimension, it was likewise determined that primary school students (*M* = 34.17, *SD* = 6.10, 95% CI [32.60–35.74]) obtained significantly higher scores than middle school students (*M* = 31.98, *SD* = 6.55, 95% CI [31.42–32.54]). For the overall “metacognitive awareness” scores, a similar linear pattern emerged where primary school students achieved the highest mean (*M* = 69.00, *SD* = 8.46, 95% CI [66.82–71.18]), while middle school students demonstrated the lowest baseline (*M* = 64.30, *SD* = 10.22, [63.43–65.17]). The partial eta squared (η$\eta_{p}^{2}$) statistics indicate that school type accounts for 0.9% to 2% of the total variance, pointing to a small operational effect size.

### 3.5 Metacognitive awareness and duration of music education (RQ3b)

Table 1 presents the One-Way Analysis of Variance (ANOVA) results executed to evaluate whether students' metacognitive awareness profiles and sub-components significantly differ based on the total duration of their music education, addressing the first educational variable within the third research question (RQ3).

As shown in Table 1, the duration of music education does not yield a statistically significant effect on students' scores in the sub-dimensions of “knowledge of cognition” [*F*_(2, 997)_ = 2.23, *p* = 0.11] and “regulation of cognition” [*F*_(2, 997)_ = 1.69, *p* = 0.18], nor on their overall “metacognitive awareness” levels [*F*_(2, 997)_ = 2.37, *p* = 0.09]. The 95% confidence intervals (CIs) for all duration categories exhibited substantial overlap, further confirming that metacognitive awareness levels remain stable regardless of the years of training. These results suggest that the length of exposure to music education, when considered in isolation, is not sufficient to explain variations in metacognitive awareness. Furthermore, the effect size estimates (η^2^ ≤ 0.005) indicate that the explanatory contribution of education duration to metacognitive differences is negligible (Ellis, 2010; Fritz et al., 2012). This theoretically motivated null finding suggests that metacognitive growth is not a passive byproduct of time, but rather a function of the quality of engagement and deliberate practice (Ericsson et al., 1993).

#### 3.5.1 Metacognitive awareness and institution type (RQ3b)

Because the null result for duration of music education could reflect either a genuine absence of a duration effect or a limitation of “years enrolled” as a proxy for substantive musical engagement, institution type (state/mandatory curriculum, *n* = 871, vs. private/fee-based course center, *n* = 124, within the analytic subsample for this test) was tested directly as a supplementary variable (Table 10). Students receiving music instruction through private course centers scored significantly higher than those receiving only state-curriculum instruction on knowledge of cognition (private *M* = 34.55, SD = 3.02; state *M* = 32.57, SD = 4.70; Welch *t*_(218.9)_ = 6.29, *p* < 0.001, *d* = 0.44, 95% CI for the mean difference [1.36, 2.60]), regulation of cognition (private *M* = 33.49, SD = 5.09; state *M* = 32.28, SD = 6.44; Welch *t*_(184.3)_ = 2.39, *p* = 0.018, *d* = 0.19, 95% CI [0.21, 2.21]), and overall metacognitive awareness (private *M* = 68.04, SD = 7.16; state *M* = 64.85, SD = 10.04; Welch *t*_(199.4)_ = 4.38, *p* < 0.001, *d* = 0.33, 95% CI [1.75, 4.62]).

**Table 10:** Comparison of metacognitive awareness by institution type (state-curriculum vs. private/fee-based music instruction; supplementary analysis).

Dimension	State *M* (SD)	*n*	Private *M* (SD)	*n*	*t* (df)	*p*	d	95% CI (diff.)
Knowledge of cognition	32.57 (4.70)	871	34.55 (3.02)	124	6.29 (218.9)	< 0.001	0.44	[1.36, 2.60]
Regulation of cognition	32.28 (6.44)	871	33.49 (5.09)	124	2.39 (184.3)	0.018	0.19	[0.21, 2.21]
Total metacognitive awareness	64.85 (10.04)	871	68.04 (7.16)	124	4.38 (199.4)	< 0.001	0.33	[1.75, 4.62]

Welch's *t*-test (unequal variances). d = Cohen's d; 95% CI is for the mean difference (private minus state). Analytic subsample for this supplementary test, *n* = 995 with valid institution-type codes.

Two follow-up chi-square tests (Table 11) clarify what this institution-type effect does, and does not, share with the duration-of-education variable reported above. First, institution type was not significantly associated with the distribution of reported duration of music education, χ^2^(6) = 12.78, *p* = 0.81: students at state and private institutions reported broadly similar numbers of years enrolled. Second, institution type was strongly associated with daily practice duration, χ^2^(4) = 285.67, *p* < 0.001: only 40.3% of students receiving private instruction reported no daily practice, compared with 78.6% of students in state-curriculum instruction. Taken together, these results indicate that “years enrolled” is a similarly coarse exposure index in both institutional contexts and therefore cannot, by itself, distinguish them, whereas institution type captures a real difference in engagement intensity (reflected in daily practice) that is associated with metacognitive awareness. This supports, with direct empirical evidence rather than assertion alone, the interpretation offered in Section 4.5 that duration of music education functions in this dataset as an administrative, exposure-based index rather than a proxy for the quality or intensity of musical engagement.

**Table 11:** Chi-square tests of association between institution type and duration of music education/daily practice duration.

Association tested	χ^2^	df	*n*	*p*
Institution type × Duration of music education	12.78	6	995	0.805
Institution type × Daily practice duration	285.67	4	995	< 0.001

Institution type: state-curriculum vs. private/fee-based instruction (Section 2.2). A non-significant association with duration of music education, alongside a strong association with daily practice duration, indicates that duration of music education is a similarly coarse exposure index across institution types, whereas institution type tracks differences in practice engagement.

### 3.6 Metacognitive awareness and type of instrument played (RQ4a)

As shown in Table 6, instrument type was associated with a statistically significant difference in the “knowledge of cognition” sub-dimension [*F*_(4, 712)_ = 8.56, *p* < 0.001, η^2^ = 0.046], with *post-hoc* comparisons indicating that students trained in string and wind instruments scored significantly higher than those trained in percussion. This effect size is small-to-moderate in magnitude, but these differences should be interpreted with caution given the small and unbalanced instrument subgroups (see Table 6 for exact ns). No statistically significant difference by instrument type emerged for “regulation of cognition” [*F*_(4, 712)_ = 1.51, *p* = 0.20]. Overall “metacognitive awareness” did show a statistically significant difference by instrument type [*F*_(4, 712)_ = 3.39, *p* = 0.01, η^2^ = 0.019], although the effect size was small; given its strong correlation with the knowledge dimension, this pattern most likely reflects the same string/wind-vs.-percussion contrast observed above, though dedicated *post-hoc* comparisons were not conducted for this omnibus test.

### 3.7 Metacognitive awareness and daily instrument practice duration (RQ4b)

Table 2 presents the Kruskal–Wallis H test results executed to determine whether students' metacognitive awareness profiles differ significantly across distinct configurations of daily instrument practice investment, addressing the primary behavioral indicator within the fourth research question (RQ4).

As shown in Table 2, daily instrument practice duration was associated with statistically significant differences in “knowledge of cognition” [χ^2^(3) = 21.76, *p* = 0.001], “regulation of cognition” [χ^2^(3) = 15.84, *p* = 0.01], and overall “metacognitive awareness” [χ^2^(3) = 22.66, *p* = 0.001]. Pairwise Mann–Whitney U comparisons with Bonferroni correction for six comparisons (adjusted α = 0.0083) indicated that the only comparison remaining statistically significant after correction was “not practicing” vs. “1 h per day” (all three dimensions, padj < 0.002; rank-biserial *r* = 0.15–0.19). Although the “2–4 h” subgroup displayed the numerically highest mean rank on all three dimensions, none of its pairwise comparisons with the other groups reached significance after correction (all padj >0.25), and the same was true for the “5+ h” subgroup (all padj = 1.00). Given this, the apparent 2–4-h peak and the subsequent decline at 5+ h—visually suggestive of an inverted-U pattern—should be regarded as a descriptive, exploratory trend rather than a statistically confirmed effect; this caution is reinforced by the very small size of both subgroups (2–4 h: *n* = 10; 5+ h: *n* = 6). The epsilon-squared effect sizes (ε^2^ ≤ 0.023) indicate a small practical magnitude overall (Ellis, 2010), consistent with the interpretation that the confirmed effect (the not-practicing vs. 1-h contrast) is modest, and that the higher-duration subgroups are too small to support firm conclusions about an optimal practice range.

As a further robustness check, the four practice-duration categories were collapsed into a binary contrast between students reporting any regular daily practice (*n* = 261) and those reporting none (*n* = 736). This simplified contrast (Table 12) confirmed significant differences on knowledge of cognition (practicing *M* = 34.00, SD = 3.72; not practicing *M* = 32.39, SD = 4.77; Welch *t*_(581.4)_ = 5.56, *p* < 0.001, *d* = 0.36), regulation of cognition (practicing *M* = 33.87, SD = 5.46; not practicing *M* = 31.94, SD = 6.50; Welch *t*_(539.1)_ = 4.66, *p* < 0.001, *d* = 0.31), and overall metacognitive awareness (practicing *M* = 67.87, SD = 8.15; not practicing *M* = 64.32, SD = 10.13; Welch *t*_(563.7)_ = 5.64, *p* < 0.001, *d* = 0.37), with all three 95% confidence intervals for the mean difference excluding zero. This binary contrast, which does not depend on the small and unbalanced higher-duration subgroups, provides the clearest evidence in this dataset that engaging in some daily practice, rather than none, is associated with higher metacognitive awareness.

**Table 12:** Comparison of metacognitive awareness between students reporting any regular daily practice and those reporting none.

Dimension	Practicing *M* (SD)	*n*	Not practicing *M* (SD)	*n*	*t* (df)	*p*	d	95% CI (diff.)
Knowledge of cognition	34.00 (3.72)	261	32.39 (4.77)	736	5.56 (581.4)	< 0.001	0.36	[1.04, 2.18]
Regulation of cognition	33.87 (5.46)	261	31.94 (6.50)	736	4.66 (539.1)	< 0.001	0.31	[1.12, 2.74]
Total metacognitive awareness	67.87 (8.15)	261	64.32 (10.13)	736	5.64 (563.7)	< 0.001	0.37	[2.31, 4.77]

Welch's *t*-test (unequal variances). d = Cohen's d; 95% CI is for the mean difference (practicing minus not practicing).

To further evaluate the four-category comparison without relying on asymptotic approximations, BCa bootstrap 95% confidence intervals (Table 13A) were computed for each subgroup's overall awareness mean: none, *M* = 64.32, 95% CI [63.60, 65.05]; 1 h, *M* = 67.79, 95% CI [66.77, 68.81]; 2–4 h, *M* = 70.60, 95% CI [65.60, 74.30]; 5+ h, *M* = 66.33, 95% CI [62.67, 70.83]. Consistent with their small sizes, the bootstrap intervals for the 2–4 h and 5+ h subgroups are three to four times wider than those for the none and 1-h subgroups, confirming that any apparent peak or decline among these subgroups is estimated too imprecisely to be interpreted as a stable effect. Two-sided permutation tests (Table 13B) for all pairwise comparisons converged with the Bonferroni-corrected Mann–Whitney results reported above: only the “none” vs. “1 h” contrast remained robust (permutation *p* < 0.001; bootstrap 95% CI for the mean difference [2.18, 4.72]), whereas all comparisons involving the 2–4-h or 5+ h subgroups had wide, zero-crossing bootstrap confidence intervals for the mean difference (e.g., 2–4 vs. 5+ h: 95% CI [−1.80, 10.00]) and did not survive correction for multiple comparisons. Finally, a Jonckheere–Terpstra test (Table 13C), which evaluates the overall ordered trend across all four practice categories jointly rather than any single pairwise comparison, indicated a statistically significant increasing trend in overall metacognitive awareness with greater practice category (J = 116,693.5, z = 4.65, *p* < 0.001), with parallel results for knowledge of cognition (z = 4.47, *p* < 0.001) and regulation of cognition (z = 3.96, *p* < 0.001). Because this test pools ranks across the full sample (*N* = 997) rather than depending on the small higher-duration subgroups in isolation, it provides more powered, assumption-light evidence for an overall positive association between practice category and metacognitive awareness than the pairwise comparisons alone, while remaining agnostic about the precise shape (linear vs. non-monotonic) of that association at the upper end of the practice-duration scale.

**Table 13:** Bootstrap and permutation-based robustness checks for daily practice duration subgroups.

Panel A. BCa bootstrap 95% confidence intervals for overall metacognitive awareness, by subgroup (10,000 resamples).				
Practice category	*n*	M	SD	BCa 95% CI
None	736	64.32	10.13	[63.60, 65.05]
1 h	245	67.79	8.02	[66.77, 68.81]
2–4 h	10	70.60	6.02	[65.60, 74.30]
5+ h	6	66.33	5.13	[62.67, 70.83]
**Panel B. Pairwise mean differences: bootstrap 95% CI and two-sided permutation-test and two-sided permutation-test *p* (100,000 resamples), overall metacognitive awareness.**				
**Comparison**		**Mean diff**.	**Bootstrap 95% CI**	**Permutation** *p*
1 h vs. None		3.46	[2.18, 4.72]	< 0.001
2–4 vs. 1 h		2.83	[−1.65, 6.92]	0.303
5+ vs. 1 h		−1.45	[−5.49, 3.01]	0.682
2–4 h vs. None		6.24	[1.83, 10.30]	0.044
5+ h vs. None		2.00	[−2.00, 6.25]	0.661
2–4 vs. 5+ h		4.30	[−1.80, 10.00]	0.249
Panel C. Jonckheere–Terpstra test for an ordered trend across the four practice categories.				
**Dimension**		**J**	**z**	***p***
Knowledge of cognition		115,958.5	4.47	< 0.001
Regulation of cognition		113,926.5	3.96	< 0.001
Total metacognitive awareness		116,693.5	4.65	< 0.001

BCa, bias-corrected and accelerated bootstrap. Permutation p-values are uncorrected for multiple comparisons; only the “None vs. 1 h” contrast in Panel B survives the Bonferroni-corrected threshold applied to the primary Kruskal–Wallis analysis (Table 2, Section 3.7). The Jonckheere–Terpstra test (Panel C) evaluates the ordered alternative None < 1 h < 2–4 h < 5+ h using the full analytic sample (*N* = 997) and does not depend on the small 2–4 and 5+ h subgroups in isolation.

### 3.8 Metacognitive awareness as a predictor of music course achievement (RQ5)

To test RQ5, whether the two metacognitive awareness dimensions predict music course achievement independently of experience-related variables, a two-step hierarchical multiple regression was conducted (Table 14). In Step 1, age, duration of music education, and daily practice duration were entered as covariates; in Step 2, the standardized knowledge of cognition and regulation of cognition scores were added as simultaneous predictors.

**Table 14:** Hierarchical multiple regression predicting music course achievement from age, duration of music education, daily practice duration, and metacognitive awareness dimensions (*N* = 1,000).

Predictor	B	SE	β	*t*	*p*
Step 1: covariates only (*R*^2^ = 0.063, *F*_(3, 996)_ = 22.23, *p*<0.001)					
Age	−0.232	0.035	−0.23	−6.64	< 0.001
Duration of music education	0.159	0.035	0.16	4.39	< 0.001
Daily practice duration	0.142	0.034	0.14	3.78	< 0.001
Step 2: + metacognitive awareness dimensions (*R*^2^ = 0.077, Δ*R*^2^ = 0.015, *F*_(change)__(2, 994)_ = 7.87, *p*<0.001)					
Age	−0.231^a^	0.035	−0.21	−6.62	< 0.001
Duration of music education	0.153	0.035	0.15	4.39	< 0.001
Daily practice duration	0.128	0.034	0.13	3.78	< 0.001
Knowledge of cognition	**0.159**	**0.041**	**0.16**	**3.88**	**< 0.001**
Regulation of cognition	**−0.064**	**0.041**	**−0.06**	**−1.57**	**0.117**

^a^Re-estimated from the raw response data (*N* = 1,000) using the Aydın and Ubuz (2010) item key (knowledge of cognition: items 1–5, 11–13; Regulation of cognition: items 6–10, 14–17); B = −0.231, SE = 0.035, β = −0.21, t = −6.62, *p* < 0.001.

As shown in Table 14 step 1 was statistically significant, *R*^2^ = 0.063, *F*_(3, 996)_ = 22.23, *p* < 0.001, with all three covariates contributing independently: age was negatively associated with achievement (β = −0.23, *p* < 0.001), while duration of music education (β = 0.16, *p* < 0.001) and daily practice duration (β = 0.14, *p* < 0.001) were positively associated. Adding the two metacognitive awareness dimensions in Step 2 produced a statistically significant increase in explained variance, Δ*R*^2^ = 0.015, *F*_(change)__(2, 994)_ = 7.87, *p* < 0.001, *R*^2^ = 0.077 for the full model. Consistent with RQ5, knowledge of cognition emerged as a statistically significant, independent predictor of music course achievement even after controlling for age and experience-related variables (β = 0.16, *p* < 0.001), whereas regulation of cognition did not (β = −0.06, *p* = 0.117). All covariate effects remained essentially unchanged in magnitude and direction between Step 1 and Step 2, indicating that the predictive contribution of knowledge of cognition is not simply proxying for experience. The overall explained variance is modest (7.7%), consistent with the many unmeasured determinants of musical achievement (e.g., innate aptitude, teacher quality, instrument-specific demands) that lie outside the scope of this study; nonetheless, this is, to our knowledge, the first evidence in this literature that a specific dimension of metacognitive awareness—knowledge of cognition rather than its behavioral regulation—is independently associated with a self-reported achievement outcome in music education, rather than only with other self-report measures. The 0.001 discrepancy between the sum of the Step 1 *R*^2^ and Δ*R*^2^ (0.063 + 0.015 = 0.078) and the reported *R*^2^ for the full model (0.077) reflects standard rounding of independently computed statistics and does not indicate a computational error.

## 4 Discussion

This section interprets the findings reported in Section 3, organized by variable, and situates them within the existing metacognition and music-education literature.

### 4.1 Correlational structure of metacognitive awareness (RQ1)

Consistent with RQ1, the two core dimensions of metacognitive awareness showed a moderate, statistically significant positive correlation (*r* = 0.59, *p* < 0.001), indicating that knowledge of cognition and regulation of cognition are related yet empirically distinguishable components, in line with Schraw and Dennison's (1994) two-dimensional model. This correlation is notably lower than the *r* = 0.72 reported between the same two subscales in the original Turkish validation sample (Aydın and Ubuz, 2010) (*N* = 589 tenth-graders) and the broadly similar *r* = 0.70–0.80 range typically reported for the MAI dimensions in general academic samples (Schraw and Dennison, 1994), suggesting that in this music-education context the two dimensions may be comparatively more dissociable, plausibly because declarative and procedural knowledge about how to practice (e.g., knowing that slow practice helps) can be acquired somewhat independently of a student's actual regulatory follow-through (e.g., consistently monitoring and adjusting one's own playing in real time). This domain-specific pattern should be treated as a preliminary observation warranting comparison with other music-education samples rather than a general claim about metacognition. The dimensions of “knowledge of cognition” (theoretical awareness) and “regulation of cognition” (performance management) are structures that support each other but also differ (see Section 1.1.2, above). The stronger correlation observed between the total “metacognitive awareness” score and the regulation dimension (*r* = 0.92) relative to the knowledge dimension (*r* = 0.85) should be interpreted with caution, as this is partly an expected mathematical consequence of the total score being calculated from items shared with the regulation sub-scale, rather than solely a substantive finding; interpreted cautiously, however, this pattern is at least consistent with the processes of “strategic action and real-time error monitoring” (Schraw and Dennison, 1994, p. 468) playing a prominent role in music education, compatible with the view that musical development is not only a technical skill but also an intensive mental management process (Miles and Shevlin, 2001, p. 115).

### 4.2 Gender differences in metacognitive awareness (RQ2a)

A notable finding of the study is the gender-based difference observed in the ‘regulation of cognition' dimension, showing a tendency toward female students. Prior research suggests that female students score higher, on average, than their male counterparts on self-regulatory processes such as planning, monitoring, and evaluation (Pintrich and De Groot, 1990). This pattern is also aligned with findings from studies on executive functions and self-regulation that report gender-related differences (Adey et al., 2007). Although causal inference is not warranted from this correlational, cross-sectional design, it may be argued that the self-regulation skills associated with musical practice are also observed in broader academic and cognitive tasks, a pattern that has been discussed in terms of “far transfer” in the literature. This is consistent with the view that music education may function as a general “mental tool” that expands cognitive capacity (Moreno and Farzan, 2015). This pattern, observed across school levels, suggests that female students in this sample may internalize self-regulation strategies at earlier stages, though this interpretation requires longitudinal verification. Furthermore, while gender-based differences were statistically significant, the small effect size (*d* = 0.14) indicates that these variations have limited practical implications. As reported in Section 3.2 (Table 7), a 2 (gender) × 3 (age band) factorial ANOVA confirmed that this gender difference does not interact significantly with age band for any of the three scores, indicating that the female advantage in regulation of cognition and overall awareness is of similar magnitude across early adolescence, mid-adolescence, and emerging adulthood, rather than being concentrated in, or driven by, any single developmental stage. This suggests that the overall development of metacognitive awareness in music education follows a largely consistent trajectory across both males and females, with individual differences likely outweighing gender-specific tendencies.

In the literature, it is stated that girls' language development and self-efficacy perceptions mature faster than those of boys (Pajares and Valiante, 1997, p. 335), and this situation is reflected in the ‘regulation' and ‘overall awareness' dimensions in fields such as music that require theoretical–practical integration. The pattern observed in overall metacognitive awareness scores is consistent with the view that multiple components of the metacognitive model defined by Schraw and Dennison (1994) may function more cohesively in female students within this sample; however, causal inference is not warranted from cross-sectional self-report data. This pattern can be interpreted as suggesting that female students may perceive music education as a more systematic academic responsibility from early ages and may manage their cognitive resources more effectively during practice (Flavell, 1979, p. 907).

### 4.3 Age-group differences in metacognitive awareness (RQ2b)

The literature on metacognitive development offers one possible lens for interpreting age-related differences, although, as a cross-sectional study, the present data speak to age-group differences within this sample rather than to developmental change over time. Academic metacognitive awareness is generally reported to become evident around ages 8–10, and some accounts link the transition to “formal operational thinking” around age 11 to a deepening of strategic planning ability (Bell et al., 2023). Other studies (Ferenc, 2016; Jauk et al., 2013) report an inverted-U pattern in the effectiveness of metacognitive interventions across school levels, which they attribute to the maturation of cognitive structures supporting self-regulation. We draw on this literature cautiously, as one possible interpretive frame among several for the age-group pattern observed here, rather than as evidence that our cross-sectional data themselves demonstrate a developmental process.

The age-related differences identified in this study are, in the first instance, age-group differences observed within this particular sample; the following paragraph offers one plausible interpretive account for this pattern, drawing on the developmental literature, while the paragraph after it shows that weekly instructional hours and educational context also contribute to these differences and should be considered alongside any developmental account. Metacognitive development, conceptualized by Flavell (1979), is characterized by the increasing complexity of “monitoring” skills during the transition from childhood to adolescence. This non-monotonic, U-shaped pattern—characterized by comparatively high scores in early adolescence (10–14 years), a marked decline during mid-adolescence (15–18 years), and a subsequent rebound in emerging adulthood (19+ years)—maps onto recognized milestones in the development of cognitive self-regulation. The elevated awareness observed among the 10–14 cohort may be tentatively associated with the strong intrinsic motivation and task engagement that often characterize early, elective music instruction (Ferenc, 2016), although the cross-sectional design of this study cannot directly test this mechanism. More notably, the decline observed among the 15–18 cohort is consistent with what has been described elsewhere as a developmental “dip” in metacognitive functioning during mid-adolescence, when shifting social-emotional demands, educational transitions, and ongoing cognitive restructuring can temporarily disrupt self-reported self-regulatory behavior (Kuhn, 2000; Veenman et al., 2006). The subsequent increase among the 19+ cohort aligns with cognitive maturation and the accumulation of deliberate learning experience, since sustained engagement with a complex domain and progressive professionalization are understood to support more advanced metacognitive monitoring and strategic planning over time (Ericsson et al., 1993). Given the cross-sectional nature of the present study, however, these accounts are offered strictly as theoretically plausible, tentative developmental pathways rather than as confirmed causal mechanisms, and they await verification in future longitudinal work.

As a robustness check on the three-band age grouping used throughout, we re-examined the age pattern using five finer, more conventional bands (10–13, 14–15, 16–18, 19–20, 21+) derived from the raw response data. The U-shaped pattern persisted at this finer resolution, with overall awareness declining from the youngest band (*M* = 69.83) through a low point in the 16–18 band (*M* = 63.42) before recovering among older students (*M* = 66.62 and 68.11 for the 19–20 and 21+ bands, respectively), *F*_(4, 995)_ = 8.11, *p* < 0.001. This finer-grained analysis confirms that the non-monotonic pattern is not an artifact of the specific three-band grouping adopted for the main analyses. We also examined whether the age and school-type effects might instead reflect differences in weekly music-instruction hours, a potential confound raised by the overlap between age, grade level, and curricular intensity. In a joint regression model entering age band, school type, and weekly hours of music instruction simultaneously (Table 8; *R*^2^ = 0.038, *F*_(5, 994)_ = 7.95, *p* < 0.001), weekly hours remained an independent, statistically significant predictor of overall awareness, B = 0.656, *t* = 2.72, *p* = 0.007, even after age and school type were controlled, while the age-band and school-type effects were attenuated (15–18 vs. 10–14: *t* = −1.46, *p* = 0.144; Middle vs. High school: *t* = 1.08, *p* = 0.280); notably, Primary school remained a significant positive predictor relative to High school, B = 4.17, *t* = 2.14, *p* = 0.032. This suggests that at least part of what appears as an age- or school-type-related difference may be attributable to differences in curricular intensity (weekly instructional hours) that covary with age and institutional level, rather than to age or school type as such; we did not include grade level in this joint model because it was highly collinear with age (*r* = 0.95, VIF > 10), which would have produced unstable estimates for both. This finding is consistent with, and reinforces, the broader interpretive caution already noted above regarding age, grade level, and institutional context in this sample.

### 4.4 School type and grade level (RQ3a)

The differences observed in the context of school type can be explained by the “effect of environmental stimuli on metacognitive development” emphasized by Veenman et al. (2006). In school types where academic expectations and curriculum intensity differ, the need for students to monitor their own cognitive performance also increases proportionally. This pattern is consistent with the view that metacognitive awareness is not merely a by-product of general intelligence, but also a contextual competence associated with the quality of education (Veenman et al., 2006). It is also possible to consider that different curriculum structures and levels of academic difficulty may be associated with higher levels of metacognitive awareness as an adaptive response to more demanding academic environments. Concretely, according to the officially mandated national curriculum (MEB, 2021), weekly music instruction time differs substantially across the school types represented in this sample: in primary schools (grades 1–4, *n* = 58) and middle schools (grades 5–8, *n* = 535), Music is a compulsory, stand-alone subject delivered for 1 lesson hour per week at every grade level; in Anatolian High Schools (*n* = 8), Science High Schools (*n* = 15), and Social Sciences High Schools (the “MAE” school in this sample, *n* = 344), Music is offered as two lesson hours per week (typically as part of a combined “Visual Arts/Music” elective in which the student selects one of the two arts); in Vocational and Technical Anatolian High Schools (*n* = 4), compulsory Music is offered only in grade 9 (2 h/week), with no compulsory music instruction in grades 10–12; and in the Fine Arts High School–Music Department (*n* = 34), music-specific professional courses (instrument training, piano, solfège, choir, orchestra/ensemble, music history, and related courses) collectively total approximately 24–26 lesson hours per week, depending on grade level. This curriculum-mandated variation in weekly instructional exposure across school types—roughly 12 to 26-fold higher in the Fine Arts High School than in general schools—is a plausible contextual driver of the observed group differences and should be taken into account when generalizing these findings beyond similarly structured educational systems.

### 4.5 Duration of music education (RQ3b)

Regarding RQ3b, the total duration of music education did not produce statistically significant differences on any sub-dimension or the overall score (all *p* > 0.05; η^2^ ≤ 0.005; see Table 1). This theoretically motivated null result is itself informative: as students move from novice to expert status, self-reflection, error detection, and strategic planning abilities generally become more developed (Li et al., 2023a; Mullens, 2025), and long-term practice is thought to install a general cycle of goal-setting, monitoring, and strategy revision that can transfer to other academic fields (Schellenberg, 2004). However, the absence of a statistically significant effect of duration in this sample suggests that metacognitive growth is not simply a passive byproduct of elapsed time, but is more closely tied to the quality and intentionality of engagement (Ericsson et al., 1993). From a cognitive load perspective (Sweller, 1988), this pattern is also consistent with the idea that technical performance occupies a large share of working memory for novice students, whereas automatization of motor skills in more experienced students frees cognitive resources for higher-level monitoring and evaluation, which duration alone does not guarantee. This distinction is conceptually important: duration of music education, as measured here, captures how long a student has been formally enrolled, a largely administrative, exposure-based index, whereas daily practice duration (Section 4.6) captures how intensively a student currently engages with the instrument, closer to what deliberate-practice theory treats as the active ingredient of skill development (Ericsson et al., 1993). The present pattern of results, a null effect for enrollment duration alongside a significant effect for daily practice intensity, is itself consistent with this distinction: it suggests that simply being enrolled longer does not by itself cultivate metacognitive awareness, whereas how a student currently practices may matter more than how long they have been nominally enrolled. This interpretation is not merely inferential: a supplementary analysis (Section 3.5.1) shows that institution type (state-curriculum vs. private/fee-based instruction) is itself significantly associated with metacognitive awareness (all *p* ≤ 0.018, *d* = 0.19–0.44), while being statistically unrelated to the distribution of reported duration of music education [χ^2^(6) = 12.78, *p* = 0.81] and strongly related to daily practice engagement [χ^2^(4) = 285.67, *p* < 0.001]. In other words, “years enrolled” appears to be an equally coarse exposure index across both types of institution, whereas the intensity of engagement that institution type partly captures, operationalized here through daily practice, is what relates to metacognitive awareness; this is consistent with, and strengthens, the administrative-index interpretation offered above.

### 4.6 Instrument type and daily practice duration (RQ4a, RQ4b)

Consistent with RQ4a, instrument type yielded a statistically significant, small-to-moderate effect on the “knowledge of cognition” sub-dimension (η^2^ = 0.046; see Table 6), with students trained in string and wind instruments scoring higher than those trained in percussion. This is plausibly related to the differing technical and cognitive demands of these instruments (e.g., the polyphonic thinking required by keyboard or string instruments compared with monophonic instruments), which may shape how students develop and differentiate their regulation strategies; given the small and unbalanced instrument subgroups in this sample, however, this pattern should be treated as preliminary.

Regarding RQ4b, only one pairwise comparison survived Bonferroni correction: students practicing about 1 h per day reported higher metacognitive awareness than those who did not practice at all. Descriptively, mean ranks were highest in the 2–4 h group and lower in the 5+ h group (Table 2), a pattern suggestive of an inverted-U shape; however, both subgroups are very small (2–4 h: *n* = 10; 5+ h: *n* = 6), and neither differed significantly from the other groups after correction. This pattern is therefore treated as an exploratory, hypothesis-generating observation, and we do not build a substantive theoretical interpretation on it (e.g., cognitive fatigue or an “optimal” practice range) without replication in a larger, more balanced sample. The more robust conclusion is the distinction between students who report regular practice and those who do not: because most participants (73.6%) reported no regular daily practice, a binary contrast was evaluated (Section 3.7, Table 12) and showed a small-to-moderate, statistically robust association with metacognitive awareness across all three scores (*d* = 0.31–0.37, all *p* < 0.001), independent of the small higher-duration subgroups (Hallam, 2001, p. 30). The ordered-trend and bootstrap checks reported in Section 3.7 (Table 13) point the same way: the existence of a positive association between practice and awareness is better supported than the precise shape of that association at the upper end of the practice scale. RQ4b's relevance thus lies primarily in the presence vs. absence of self-directed practice rather than in fine gradations of duration.

### 4.7 Metacognitive awareness as a predictor of music course achievement (RQ5)

Consistent with RQ5, knowledge of cognition emerged as a statistically significant, independent predictor of students' self-reported music course achievement (β = 0.16, *p* < 0.001), even after age, duration of music education, and daily practice duration were statistically controlled, whereas regulation of cognition did not (β = −0.06, *p* = 0.117). Because the achievement outcome is a five-band ordinal self-report rather than a truly continuous measure, we re-estimated this model using ordinal logistic regression as a sensitivity analysis; the pattern of results was unchanged, with knowledge of cognition remaining a statistically significant predictor (*p* < 0.001) and regulation of cognition remaining non-significant (*p* = 0.097), indicating that the finding is not an artifact of treating the ordinal outcome as continuous. This asymmetry is theoretically informative: it suggests that the declarative and conditional knowledge component of metacognitive awareness—knowing which practice strategies are effective, and when and why to apply them—carries predictive value for self-reported achievement that is not reducible to the behavioral act of regulating one's practice, nor to sheer accumulated experience. One plausible interpretation is that knowledge of cognition functions as a resource that is drawn upon relatively consistently once acquired (e.g., understanding why slow practice consolidates a passage), whereas regulation of cognition, being an in-the-moment behavioral process, may be more context-dependent and therefore less reliably captured by a single retrospective self-report measure, weakening its observed association with a distal achievement outcome. This finding also addresses a criterion-related validity gap in the literature: metacognitive awareness, and specifically its knowledge dimension, is associated with a self-reported achievement outcome and not merely with other self-report constructs. At the same time, the modest total variance explained (*R*^2^ = 0.077) indicates that metacognitive awareness accounts for only a small share of the many determinants of musical achievement—including factors not measured here, such as innate aptitude, instructional quality, and instrument-specific technical demands—and this finding should be interpreted as an initial, correlational demonstration of predictive relevance rather than evidence of a causal or pedagogically sufficient mechanism. Experimental or longitudinal designs that manipulate or track metacognitive knowledge directly, and that employ standardized rather than self-reported performance measures, are needed before this finding can inform instructional practice with confidence.

## 5 Conclusion

Overall, students receiving music education in this sample reported moderately levels of metacognitive awareness, encompassing both the knowledge and regulation dimensions, though this awareness did not increase monotonically with age or educational level. Five findings are of particular note. First, knowledge of cognition and regulation of cognition emerged as related but empirically distinguishable components (*r* = 0.59), consistent with Schraw and Dennison's (1994) two-dimensional model; the stronger correlations of each sub-dimension with the total score (*r* = 0.85 and *r* = 0.92) partly reflect item overlap and should not be over-interpreted as independent validity evidence. Second, gender was one of the most consistent correlates observed, with female students reporting higher awareness, particularly in the regulation dimension, although the associated effect sizes were modest. Third, metacognitive awareness differed across age groups in a non-monotonic, U-shaped pattern, with comparatively lower scores in the 15–18 band relative to both younger and older groups; this is best understood as an age-group difference observed within this sample, to which weekly instructional hours and educational context also contribute, rather than as direct evidence of a developmental process. Fourth, the most robust finding regarding practice was a binary one: students who reported any regular daily practice scored significantly higher in metacognitive awareness than those who reported none (*d* = 0.31–0.37, all *p* < 0.001). A numerically non-linear pattern among practicing students, with the 2–4 h subgroup reporting the highest mean ranks, is broadly consistent with the deliberate practice framework (Ericsson et al., 1993); however, this finer pattern did not survive Bonferroni correction for multiple comparisons and is based on very small subgroups (2–4 h: *n* = 10; 5+ h: *n* = 6), and should therefore be treated as an exploratory, hypothesis-generating observation rather than a confirmed finding, warranting replication in a larger, more balanced sample before any claims about an “optimal” practice duration are drawn.

Fifth, and moving beyond purely descriptive correlates, a hierarchical regression testing the predictive relevance of metacognitive awareness for students' self-reported music course achievement showed that knowledge of cognition, but not regulation of cognition, remained a statistically significant predictor after age, duration of music education, and daily practice duration were controlled (β = 0.16, *p* < 0.001, Δ*R*^2^ = 0.015). This provides initial criterion-related evidence that metacognitive awareness—specifically its knowledge dimension—is associated with a self-reported achievement outcome rather than only with other self-report constructs, though the modest variance explained (*R*^2^ = 0.077) indicates that metacognitive awareness is one of many contributors to musical achievement rather than a dominant one.

Taken together, these findings suggest that metacognitive awareness in music education is shaped less by the sheer duration of training than by the gender, age, and practice-related characteristics of the learner. They support the broader view that music education functions not only as artistic training but also as a context for developing general self-regulatory skills and metacognitive awareness, with implications for how practice and instruction are structured across different stages of music education.

## 6 Limitations and suggestions for future research

Although this study comprehensively examines the metacognitive awareness of music students in a large sample ranging from primary school to high school, several methodological and contextual limitations should be considered when interpreting the findings. Sample and generalizability: the research data are limited to primary, middle, and fine arts high school students studying in specific regions of Türkiye. Although the sample size (*N* = 1,000) is sufficient in terms of statistical power, the results cannot be directly generalized to music students in other socio-cultural contexts or to students at different institutional levels (e.g., university, conservatory). In addition, several grouping variables include very small subgroups, most notably at the upper end of daily practice duration, for specific instrument categories, and for the Fine Arts High School subgroup within school type (see Tables 1–6 for exact subgroup sizes); statistical comparisons involving these categories should be interpreted with caution, as small cell sizes reduce statistical power and increase the risk of unstable estimates.

Population heterogeneity: the great majority of participants (87.1% within the analytic subsample; Section 2.2) received music instruction through the compulsory national curriculum in general-education schools rather than through specialized or audition-based programs, and 73.6% reported no regular daily instrument practice (Table 15). Throughout this article, “general school music education” therefore refers to compulsory classroom instruction, and “private or specialized instruction” to fee-based course-center training; the study describes metacognitive awareness across this full range rather than among intensively engaged, pre-professional musicians. This heterogeneity is also informative: institution type and the presence of daily practice were each significantly associated with metacognitive awareness (Sections 3.5.1 and 3.7). Findings involving daily practice (RQ4a, RQ4b) should accordingly be read as describing this broad, school-based population and not extrapolated to populations selected for intensive musical training.

**Table 15:** Sociodemographic profile of the participating students.

Demographic information	Category	*f*	%
Gender	Female	578	57.8
	Male	420	42.0
	Unspecified^a^	2	0.2
	Total	1,000	100
Age	10–14	119	11.9
	15–18	586	58.6
	19+	295	29.5
	Total	1,000	100
Type of school attended	Primary	58	5.8
	Middle	535	53.5
	Anatolian high school	8	0.8
	Fine arts high school	36	3.6
	Social sciences high school	344	34.4
	Vocational and technical Anatolian high school	4	0.4
	Science high school	15	1.5
	Total	1,000	100
Duration of music education	1 year	346	34.6
	2–4	363	36.3
	5–6	227	22.7
	7+	64	6.4
	Total	1,000	100
Instrument	Not specified	283	28.3
	Keyboard	281	28.1
	String	205	20.5
	Wind	123	12.3
	Bowed string	56	5.6
	Percussion	52	5.2
	Total	1,000	100
Daily practice time	None	737	73.7
	1 h	247	24.7
	2–4 h	10	1.0
	5+	6	0.6
	Total	1,000	100

^a^Two participants did not select a valid category on the gender item and are reported separately as “Unspecified” rather than being merged into either category; these two cases were excluded from all gender-based analyses (Table 3), which accordingly report *N* = 420 (male) and *N* = 578 (female), df = 996.

Measurement tool limitation: the data were collected through a self-report scale reflecting students' own perceptions. In measuring internal processes such as metacognitive awareness, it is a limitation that participants, especially younger students (primary school), may tend to present themselves as more strategic than they actually are, due to the effect of “social desirability” (Schraw and Dennison, 1994, p. 468). Furthermore, while the psychometric properties of the Metacognitive Awareness Inventory (MAI) were supported by high internal consistency coefficients in the present sample (α total = 0.84, α knowledge = 0.76, α regulation = 0.81), this study did not include a confirmatory factor analysis (CFA) to verify the two-dimensional structure empirically within this specific cohort. Although the scale's structural validity has been previously established in the Turkish context (Aydın and Ubuz, 2010), the absence of a sample-specific structural check remains a methodological limitation, and future research should employ structural equation modeling (SEM) or CFA to confirm whether the knowledge and regulation dimensions maintain their theoretical distinctiveness across different levels of music education. A related limitation concerns the age-appropriateness of the instrument: the MAI was originally developed and validated with adolescent and adult populations, and its Turkish adaptation (Aydın and Ubuz, 2010) was likewise validated with older students; whether its items are equally comprehensible and psychometrically equivalent for the youngest participants in the present sample (primary school students as young as 10–14 years) has not been separately established, and reading-comprehension or self-reflection demands may not be uniform across the full age range studied here (approximately 10–20 years, with a small subset reporting older ages). Future work should evaluate measurement invariance of the MAI across these age bands before treating scores as directly comparable. This caveat applies directly to the age-group comparisons reported in Sections 3.3 and 4.3: in the absence of a confirmed, invariant factor structure across age bands, differences between age groups should be interpreted as observed differences in mean scores on this instrument, rather than as unambiguous evidence of differing levels of an identically structured underlying construct.

Methodological limitation: the study was conducted using a cross-sectional design, which reflects the current state at a specific point in time rather than tracking developmental changes in metacognitive awareness (such as the transition from primary school to high school) over time. The lack of longitudinal data limits the ability to determine cause–effect relationships between variables (Tabachnick and Fidell, 2013, p. 79).

Scope of variables: the study focused on a defined set of variables, namely gender, educational level, type of instrument, and practice duration; other external and affective variables that may affect students' metacognitive awareness were outside its scope. Because metacognitive monitoring is inherently self-referential—requiring students to represent and evaluate their own cognitive activity—it is also situated within broader models of self-processing, agency, and identity (Di Plinio et al., 2020). Future research may benefit from integrating measures of musical identity, self-efficacy, and motivational orientation alongside metacognitive awareness instruments.

Performance observation: the study measured students' metacognitive knowledge and regulation through a self-report scale rather than through direct performance observation or video analysis of the extent to which students actually apply these strategies during instrument performance (Zimmerman, 2000, p. 14). Although Section 3.8 extends the design beyond purely descriptive correlation by testing whether metacognitive awareness predicts students' self-reported music course achievement, this achievement measure is itself a coarse, five-band self-report of school grades rather than a standardized, independently verified performance assessment (e.g., a jury-rated technical or musical proficiency test), and is therefore subject to the same self-report limitations, including possible social-desirability bias, as the metacognitive awareness measure itself. Consequently, the present study remains correlational rather than causal in design; future research should ideally employ longitudinal designs incorporating standardized, externally rated musical performance assessments to clarify and strengthen the predictive relationship between metacognitive awareness and objective musical performance identified here.

### 6.1 Recommendations for practice

Considering that music education and metacognitive awareness appear to be reciprocally related, and that a high level of metacognitive awareness is valuable for all students and not only those receiving music education, increasing extracurricular music activities and encouraging instrumental engagement as a hobby—even outside professional or academic tracks—may support the development of general cognitive processes (Şendurur and Akgül Bariş, 2002). Because male students in this sample showed comparatively lower averages in the regulation of cognition dimension, targeted “learning to learn” and self-monitoring workshops for this group may be particularly beneficial, alongside broader “metacognitive guidance” introduced from the primary school level onward to help students become aware of their own thinking processes from an early age.

### 6.2 Recommendations for future research

Future studies should follow the same cohort of students longitudinally throughout their music education to examine how metacognitive awareness changes over time, and should consider diversifying research on the neurological foundations of the relationship between music education and metacognitive capacity. In addition, because related motivational and technological constructs such as self-efficacy and artificial-intelligence readiness have been shown to jointly account for a substantial share of variance in music students' academic performance (Wang and Li, 2024), future research could usefully examine how metacognitive awareness relates to these constructs within the same models.

## Data Availability

The original contributions presented in the study are included in the article/supplementary material, further inquiries can be directed to the corresponding author.
